# Remote Nanoscopy with Infrared Elastic Hyperspectral Lidar

**DOI:** 10.1002/advs.202207110

**Published:** 2023-03-25

**Authors:** Lauro Müller, Meng Li, Hampus Månefjord, Jacobo Salvador, Nina Reistad, Julio Hernandez, Carsten Kirkeby, Anna Runemark, Mikkel Brydegaard

**Affiliations:** ^1^ Department of Physics Lund University Sölvegatan 14c Lund 22363 Sweden; ^2^ Centre for Environmental and Climate Science Lund University Sölvegatan 37 Lund SE‐223 62 Sweden; ^3^ Norsk Elektro Optikk A/S Østensjøveien 34 Oslo 0667 Norway; ^4^ Department of Veterinary and Animal Sciences Copenhagen University Frederiksberg 1870 Denmark; ^5^ FaunaPhotonics Støberigade 14 Copenhagen 2450 Denmark; ^6^ Department of Biology Lund University Sölvegatan 35 Lund 22362 Sweden

**Keywords:** biophotonics, hyperspectral imaging, infrared spectroscopy, lidar, supercontiuum, thin film physics, insects

## Abstract

Monitoring insects of different species to understand the factors affecting their diversity and decline is a major challenge. Laser remote sensing and spectroscopy offer promising novel solutions to this. Coherent scattering from thin wing membranes also known as wing interference patterns (WIPs) have recently been demonstrated to be species specific. The colors of WIPs arise due to unique fringy spectra, which can be retrieved over long distances. To demonstrate this, a new concept of infrared (950–1650 nm) hyperspectral lidar with 64 spectral bands based on a supercontinuum light source using ray‐tracing and 3D printing is developed. A lidar with an unprecedented number of spectral channels, high signal‐to‐noise ratio, and spatio‐temporal resolution enabling detection of free‐flying insects and their wingbeats. As proof of principle, coherent scatter from a damselfly wing at 87 m distance without averaging (4 ms recording) is retrieved. The fringed signal properties are used to determine an effective wing membrane thickness of 1412 nm with ±4 nm precision matching laboratory recordings of the same wing. Similar signals from free flying insects (2 ms recording) are later recorded. The accuracy and the method's potential are discussed to discriminate species by capturing coherent features from free‐flying insects.

## Graphical abstract

A new elastic hyperspectral Scheimpflug lidar that can simultaneously resolve flashes from insect wings in range, time, and wavelength is demonstrated. The coherent scattering from a distant museum insect wing and also from free‐flying insects is captured. The fringed backscattered echo is explained by a thin‐film model enabling determination of the wing thickness with a precision of a few nanometers.

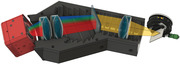

## 1 Introduction

Insects decline, in both abundance and diversity, at alarming rates.^[^ ^1^, ^2^, ^3^, ^4^ ^]^ This loss of insect poses a major threat to the ecosystem services they provide, including crop pollination.^[^ ^5^ ^]^ The main reasons have been identified as habitat loss and the use of pesticides.^[^ ^6^ ^]^ Conventional insect inventorying is accomplished by trapping,^[^ ^1^, ^7^ ^]^ and the subsequent analysis is both time‐consuming and expensive. To speed up this process, innovative approaches are needed for insect surveillance. Machine vision has been applied in field,^[^ ^8^ ^]^ on traps,^[^ ^9^ ^]^ and for trap catch analysis.^[^ ^10^ ^]^ Distributed sensors picking up the wingbeat oscillations from insects are also reported.^[^ ^11^, ^12^ ^]^ The fact that free‐flying insects appear sparsely in time and space,^[^ ^13^ ^]^ implies that insect monitoring becomes particularly efficient when the probe‐volume is elongated over hundreds of meters and trans‐illuminated by a laser beam.^[^ ^14^, ^15^ ^]^ Hereby, an enormous amount of insects can be observed daily using only a couple watts of light as opposed to hyperspectral push broom imaging,^[^ ^16^ ^]^ where hundreds of Watts illumination is split up between every pixel footprint. Hyperspectral lidar can be implemented with only a few Watts of illumination as the same laser light is recycled from one pixel footprint to the next one until it intercepts an insect or the end of the transect. The wide range of detection distances and the rapid movements of free flying insects, however, cause focus challenges and motion blur in spatial domain imaging. Alternatively, insects can be classified in the frequency domain by their wingbeat frequency and overtone spectra^[^ ^17^, ^18^ ^]^ which is not subject to optical defocusing. Wingbeats range from 10 to 1000 Hz, but the relative frequency spread within a single species and sex are generally some 20–40% broad^[^ ^19^ ^]^ and depends on temperature^[^ ^15^ ^]^ and body mass variations^[^ ^20^, ^21^ ^]^ (e.g., age, reproductive stage, or pollen payload). In particular, the 100–200 Hz region is crowded with species, and the frequency domain alone is unlikely to be able to differentiate the thousands of species in a single habitat.

Adding spectroscopy to lidar allows retrieval of tiny features over far distances; for example, picometer spacings between electron shells in atoms^[^ ^22^ ^]^ or molecules.^[^ ^23^ ^]^ Micrometer features such as snow grain size or eggs inside a mosquito abdomen can be deduced from incoherent scattering by measuring the equivalent water absorption pathlength^[^ ^24^ ^]^ or depolarization ratio.^[^ ^20^ ^]^ Insects have either two or four wings, which constitute thin films,^[^ ^25^, ^26^ ^]^ and depending on the membrane thickness, the wings can exhibit resonant backscatter for specific wavelengths. These wing interference patterns (WIPs) have been proposed to be important for mate choice.^[^ ^27^, ^28^ ^]^ Such mate choice signals are expected to be highly species‐specific.^[^ ^29^ ^]^ The spectral fringes from wings can be preserved during spatial integration across the wing, producing a fringed spectrum from the whole wing.^[^ ^30^ ^]^ The two or four wing surface normal visits a large fraction of the hemispheres during each wingbeat, and when a wing surface normal coincides with the source‐detector midpoint, a coherent flash appears. This microsecond instance represents one of the rare occasions where the orientation of the insect's wing is known, and an accurate assessment of area, thickness, and reflectance can be done. In a previous study, two closely related mosquito species could be differentiated by such flash chromaticity,^[^ ^19^ ^]^ although the species were impossible to differentiate by morphology in the microscope. Wing flashes have previously been retrieved remotely by polarization lidar^[^ ^28^ ^]^ and dual‐band lidar.^[^ ^31^ ^]^ These studies have eluded on the existence of coherent scattering, but until now no remote instrumentation has allowed to capture such flashes, resolve them spectrally and uniquely determine the wing thickness.

In this work, we demonstrate a new elastic hyperspectral Scheimpflug lidar (EHSL) that can simultaneously resolve flashes from insect wings in range, time, and wavelength. The approach is based on continuous wave Scheimpflug lidar^[^ ^23^, ^32^ ^]^ and its inelastic hyperspectral variety, which was previously developed in fluorescence mode.^[^ ^33^, ^34^, ^35^ ^]^

## 2 Results

### 2.1 EHSL

**Figure 1:**
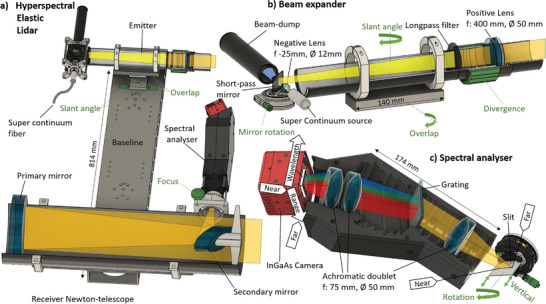
Elastic hyperspectral Scheimpflug lidar (EHSL). Color coding; gray: optomechanics, green: degrees of freedom for alignment, yellow: broadband light cones. a) The beam expander and receiver mounted on a Scheimpflug lidar base of 81 cm. b) Light from a supercontinuum source is stripped from visible light, expanded and collimated. c) Retrieved light is dispersed in photon energy perpendicular to the slit. Both the slit and the 2D InGaAs array are tilted according to the Scheimpflug condition and the hinge rule.

Based on previous Scheimpflug lidars, we developed a system with sufficient sensitivity, range resolution, spectral range as well as the necessary speed to potentially capture a flash from a free flying insect wing. Here, a baseline bar (**Figure** **1**a) separates the beam expander and receiver. In order to vastly increase the number of spectral bands, a broadband supercontinuum light source (stripped from its visible emission) is used, expanded and transmitted to the atmosphere (Figure 1b). In the tilted focal plane of the receiving telescope, backscattered light passes a slit and is dispersed in photon energy by a 3D printed spectral analyzer (Figure 1c). The backscattered and dispersed light is projected on a 2D InGaAs camera where 1D represents target distance and the other dimension represent wavelength.

### 2.2 Retrieved Spectral Fringe

**Figure 2:**
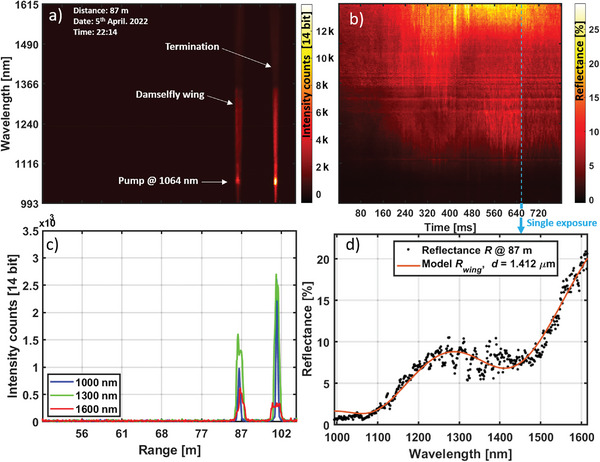
Remote Nanoscopy. a) Instantaneous lidar range‐wavelength map (light distribution on the 2D InGaAs array), the signal is uncalibrated intensity counts and the Nd:YAG supercontinuum pump laser is seen. b) Calibrated lidar time‐wavelength reflectance map at 87 m range made from 200 consecutive exposures. A single, representative, exposure/echo (marked by blue arrow) is selected for further inspection. c) This lidar echo at three selected wavelength bands, the wing displays distinct chromaticity compared to the spectrally flat neoprene termination. d) Retrieved reflectance spectrum from a damselfly wing at 87 m range acquired during 4 ms compared to a thin film model. At this instance and wing orientation, the thickness is estimated to 1412 ± 4 nm (95% confidence interval and 96% *R* ^2^_adj_ ).

To demonstrate the capacity of rapid retrieval and resolving an insect wing and quantifying the thickness, we inserted a forewing of a female damselfly, *Calopteryx virgo*, into the lidar transect at 87 m range (**Figure** **2**a). The echo from the beam termination on a black neoprene target is also seen at 100 m range. The data were collected at a pace of 200 Hz and exposure times of 4 ms (Figure 2b). When the wing surface orientation is in specular conditions, high signal magnitude is observed. Such single unfiltered exposure from the lidar is displayed in Figure 2c. The hyperspectral echo from the delicate wing displays a spectral fringe Figure 2d. We could accurately model the remotely retrieved fringe by thin film theory^[^ ^26^ ^]^ and determine the thickness with an astonishing accuracy of a few nanometers corresponding to less than 1% relative error.

### 2.3 Hyperspectral Imaging

**Figure 3:**
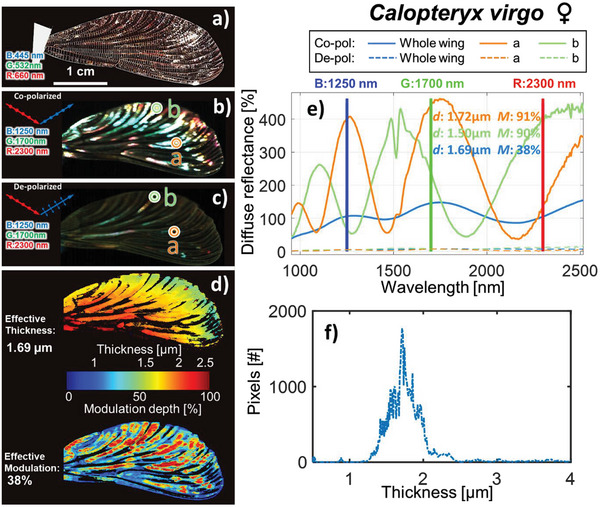
Laboratory recordings. a) A damselfly wing appears glossy under a specular illumination; structural colors can be seen on some wing spots. b,c) False color image of the same wing in co‐ and depolarization (band choices: blue @ 1250 nm, green @ 1700 nm, and red @ 2300 nm) by infrared hyperspectral imaging. d) Map of the wing thickness and modulation depth. e) Examples of fringes of selected orange and green circled wing pixel from the thin and thick region as well as the effective fringe (blue line) from the whole wing. The thickness, *d*, and the modulation depth, *M*, is indicated. The absence of depolarized reflectance indicates that the phenomenon is highly coherent. f) Histogram of thicknesses across the examined wing.

To validate the thickness value and the precision, we analyzed the same wing (**Figure** **3**a) in detail in the laboratory by polarimetric hyperspectral imaging^[^ ^36^ ^]^ (Figure 3b,c). A spectral fringe model^[^ ^26^ ^]^ was applied to the individual pixels, and the thicknesses were estimated across the wing surface (Figure 3d). The wing contains cells with thin and thick membranes but also an effective fringe for the wing as a whole (Figure 3e). The absence of fringes in the depolarized recordings verifies that the phenomena are coherent, and a high degree of collimation from the backscattered light can be expected. Thus, values exceed 100% compared to a diffuse Lambertian reflectance standard. The encountered thickness across the wing is displayed in Figure 3f. The remotely retrieved fringe was recorded without the knowledge of the orientation of the wing (as would be the case for free‐flying insects). Thus, only a specific part of the wing could fulfill the specular reflection criterion in the field experiment. However, the remotely measured thickness of 1.41 µm closely resembles the effective thickness which is 1.69 µm and falls within the thickness encountered across the wing.

### 2.4 Lidar Signals from Free Flying Insects

**Figure 4:**
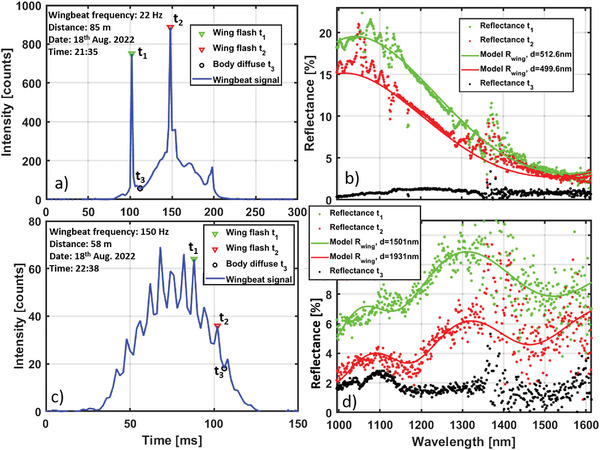
Examples of hyperspectral lidar signals from unknown free flying insects. a) Average intensity of all spectral bands versus time from an insect at 85 m range. The spiky and asymmetric waveform indicates clear glossy wings and the low wingbeat frequency is typical for large insects. b) Spectral reflectance from the observation in 4a at the three indicated instances. The fringe model attained *R* ^2^_adj._ of 97% and thickness certainly of ± 2 nm (95% confidence intervals). Similarly, the consecutive flashes display 13 nm discrepancy, the body reflectance between the flashes displays a characteristic attenuation by melanin toward the shorter wavelengths. c) Another and smaller insect observed at 58 m distance where wing beat flashes are barely resolved. d) For the case in (c), the fringe model attained *R* ^2^_adj._ of only 80% and thickness certainty of ±10 nm, but the two fringes display discrepancy of 430 nm, even the body spectrum displays traces of fringed properties.

After the initial test described above, the lidar was deployed again at the same location and the experiment was left unperturbed to capture free flying insects. Numerous hyperspectral lidar observations were captured during an evening. Two representative examples are displayed in **Figure** **4**. In the left figures, the total backscattered intensity is plotted over time. Two instances of specular flashes from the wings were selected for spectral analysis. We also present a negative control in‐between the flashes, this signal arises from the insect body and should not display thin‐film fringes.

The first example in Figure 4ac was detected after sunset at 85 m distance. Multiple signals of this type were recorded with very low wing beat frequencies in the range 16–22 Hz (indicating a large insect). Further, the waveform in Figure 4a displays narrow spikes and consequently high intensity skewness. This indicates an insect species with clear, glossy wings, and a wing membrane thickness fulfilling resonant backscatter. This wing membrane is very thing and indeed the two consecutive flashes for the first example show a discrepancy of only 13 nm, see Figure 4b. The spectra from the insect body are characterized by melanin absorption toward the shorter wavelengths.

The second selected example in Figure 4c,d, is also nocturnal but a much smaller insect (intensity counts are lower despite closer range). The wingbeat of 150 Hz is marginally resolved in time, it is therefore not possible to deduce exactly how rapid and how intense the flashes are. Even so, spectral fringes could be retrieved, see Figure 4d. Despite the smaller size, the wing is three times thicker than in the previous case, however, the discrepancy is now 430 nm which is considerable. Also, the body contribution shows traces of fringed properties, this points to the fact that neither the peaks nor the valleys in the backscattered waveform were resolved properly in time.

### 2.5 Hyperspectral Lab Inspection of Possible Candidates

**Figure 5:**
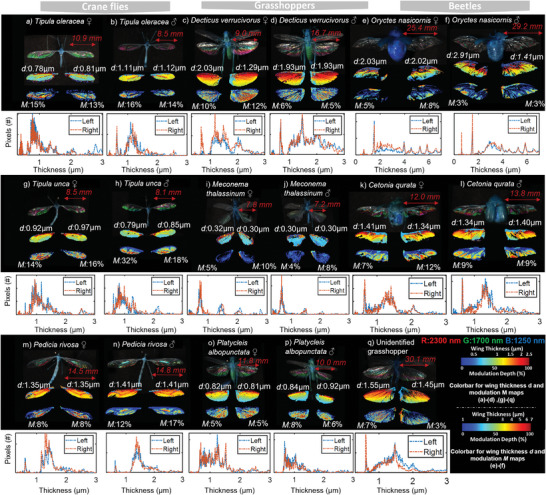
Small survey of species groups; crane flies, grasshoppers, and beetles which could possibly account for lidar signals in Figure 4. For each species and sex; top: Short‐wave infrared false color images (*R* = 2300 nm, *G* = 1700 nm, *B* = 1250 nm), middle: thickness distributions across left and right wings. The numbers, *d*, indicate the effective wing thicknesses for left‐ and right wings, bottom: wing maps indicating spectral modulation, *M* indicates fringe modulation from the entire wings. The thickness distributions for left and right wings are displayed under each specimen. (The narrow spikes in the thickness distributions should be disregarded, since these are from the veins where the algorithm could not identify a fringe).

We speculated on several insect species‐ or family candidates which could account for the free flight lidar recordings in Figure 4ab. The candidate is probably a large nocturnal insect species with ultrathin clear wings and wing beats of 16–22 Hz. Crane flies, grasshoppers, and beetles were spotted at the field site during our lidar work. From these groups, three locally common species were selected from the zoological museum. Hyperspectral image analysis was carried out as previously, **Figure** **5**. There are ≈60 insect families present at the ecological field station in late summer and much more species. Although, we encountered both crane flies and grasshoppers with similar submicron effective wing thicknesses, none of the selected species matches the recording exactly.

## 3 Discussion

This progress and the EHSL method open up for a new and complementary domain for detecting and classifying free‐flying insects in situ, namely the remote acquisition of wing thicknesses with nanometers precision. We have demonstrated that the method can be deployed in field and capture signals from insects in flight. We have shown the potential in terms of precision and also that the technique could benefit from increased sample rates. Although we could not find a perfect match to our field recordings, lidar signals could in principle be compared to pinned specimens from ecological museums. Until now, lidar techniques for detecting free‐flying insects have relied mostly on frequency analysis of wingbeat patterns, which requires multiple wingbeats during the beam transit. The EHSL technique could in principle determine the wing thickness from a single microsecond flash. This could ease constrains on beam width and power allowing to detect smaller insects at further distances, however, with a reduced probe volume. The fact that spectral fringes from insect wings exist, that they can be retrieved over distance and that these fringes are highly copolarized, specular, and coherent—also implies that their backscatter is collimated to some extent. Therefore, the range attenuation of fringed signals is likely to be weaker than the ordinary square factor known from aerosol and molecular lidar.^[^ ^37^ ^]^ Rather related studies suggest that flat lidar targets can cause back‐propagating laser light which is partially collimated.^[^ ^38^ ^]^ This could imply considerable advantages in signal‐to‐noise ratio if the sample rate increases (despite less illumination energy per exposure).

The presented recordings were carried out at night as our current supercontinuum source does not allow modulation. However, inelastic hyperspectral lidar has been demonstrated in sunlight^[^ ^35^ ^]^ (despite the weaker target interaction), thus this elastic method can be expected to work in sunlit conditions if modulation technicalities are overcome. The thin‐film effect does generally not apply to the important and large order of *Lepidoptera* (Greek: scale wings) comprising butterflies and moth. However, species in this order can be classified in the shortwave infrared region according to the surface roughness of the microstructures on their wing scales.^[^ ^36^ ^]^ The same instrument presented here also enables quantification of such scale microstructures and also the equivalent absorption path lengths of melanin^[^ ^36^ ^]^ and liquid water^[^ ^39^ ^]^ in the body. Resulting parameters from scale microstructures and equivalent absorption path lengths can also be determined quantitatively and reported in microns for inter‐comparability between studies. In fact, the detailed short‐wave infrared reflectance of insect bodies can potentially enable differentiating insect species, sexes, age groups, life stage, and infection status.^[^ ^40^, ^41^, ^42^, ^43^ ^]^ Therefore, the described technique has the potential to revolutionize insect surveillance. We foresee that other disciplines of optical remote sensing and environmental monitoring also could benefit from the outlined hyperspectral lidar method. For example, hyperspectral lidar for vegetation canopies^[^ ^34^, ^44^ ^]^ could improve tree species classification and report on leaf moisture, fertilization, or internal leaf structures. The method could also be employed in atmospheric sensing of particles^[^ ^45^, ^46^ ^]^ and gasses such as CH_4_, O_2_, H_2_O,^[^ ^47^ ^]^ and CO_2_.^23^

## 4 Experimental Section

### Field Measurements

An initial field campaign was conducted at Stensoffa field station^[^ ^48^ ^]^ in southern Sweden (55°41′44″N 13°26′50″E) on the 5th of April 2022 to test the hyperspectral lidar. A map of the experimental site is shown in Figure S1 (Supporting Information). The lidar was mounted on a tripod, pointing at a neoprene termination at 100 m distance. A near infrared silicon monitor camera was used to align and verify the broad infrared beam which is invisible to the human eye. The measurements were conducted during the night to reduce background illumination. The acquisition rate was set to 200 Hz with maximal laser power. During that measurement, a single damselfly wing was held in the beam on a blackened wire at 87 m distance. The surface orientation was varied randomly, while the system was collecting the data.

In 18th August 2022, the same site was returned for the purpose of collecting signals from free flying insects. The lidar transect was identical, but the acquisition rate was set to 500 Hz again with the maximal available power.

### Instrumentation

The lidar in this work is based on the Scheimpflug method, applying the Scheimpflug and Hinge rules which are described and developed.^[^ ^49^, ^50^, ^51^ ^]^ Mathematical derivations of the Scheimpflug principle are found.^[^ ^52^, ^53^ ^]^ In hyperspectral lidar, the Scheimpflug and Hinge rules are applied twice since not only range but also spectrum has to be determined. This concept was derived from previous studies.^[^ ^33^, ^34^, ^35^ ^]^

The lidar setup is shown in Figure 1 and consist of several elements described below:

Baseline: The receiver telescope and the beam expander are mounted on a baseline with 814 mm separation (Figure 1a). The length of the baseline, together with the angle between beam expander and telescope, and the orientation of the slit, determine the first Scheimpflug and Hinge rule.

Super continuum light source: Supercontiuum light is broadband light that is generated from picosecond pulses from a mode‐locked, diode‐pumped solid‐state laser (DPSS) that propagates through a single mode nonlinear fiber.^[^ ^54^ ^]^ The source used in this work is a Supercontiuum fiber laser (SC400 Fianium, Nordiske Kabel og Trådfabriker, Denmark) with a spectral range from 400 to 2400 nm. The wavelength of the pump is at 1064 nm, generated by a DPSS Nd:YAG laser with a repetition rate of 80 MHz, a pulse duration of 400 fs and a max pulse energy of 50 nJ. The output collimator of the supercontinuum fiber is connected to the beam expander (Figure 1a,b).

Beam expander: The purpose of the beam expander (Figure 1b) is to expand the beam of the supercontinuum source and strip off the visible part of the spectrum. The beam expander is designed with a Galilean telescope composition where first a negative lens (*f* = −25 mm, Ø = 12 mm, LC1054‐B‐ML, Thorlabs, USA) is used to expand the beam and afterward collimate it with a positive lens (*f* = 400 mm, Ø = 50 mm, ACT508‐400‐B‐ML, Thorlabs, USA). The visible part is stripped off with a dichroic mirror (SP 650 nm, 25 × 36 mm^2^ Thorlabs, USA) and a long‐pass filter (RG850 Schott, Ø = 50 mm, Edmund Optics, UK), resulting in an emitted spectral range of 850–2400 nm and an average power of ≈3 W. The beam expander is mounted on a tangential stage (Stronghold, Baader Planetarium, Germany) with which the slant‐ and overlap angles^[^ ^55^ ^]^ can be adjusted. The supercontinuum light beam is eye‐safe for aerofauna but not for humans, the experiment area was restricted for public access.

Receiver: The backscattered light is collected with a *f* = 800 mm, Ø = 200 mm Newton telescope (Quattro, SkyWatcher, China) mounted on the lidar baseline. The collected light is then focused onto the 45° tilted entrance slit of the spectral analyzer which is directly mounted in the focal stage (Figure 1ac). This fulfills the first Scheimpflug condition (Beam‐Newton‐Slit). Furthermore, the focuser of the Newton telescope can be adjusted to fulfill the hinge rule, see Figure 1a.

Spectral analyzer: The purpose of the spectral analyzer (Figure 1c) is to disperse light from the slit according to photon energy and project it on a 2D detector array, while keeping the range information along the slit intact. The aperture of the slit is 20 mm × 200 µm. A second set of Scheimpflug condition and hinge rule is considered (Slit‐Grating‐Array). The Scheimpflug condition and the hinge rule is based on simple geometrical optics considerations and assume thin optics with large F/# and no aberrations. The optics is F/4, matching the receiving Newton telescope, therefore it includes 3 lenses with several centimeters path lengths in solid glass. Because of this, the Scheimpflug condition and hinge rule can only be used as a first order approximation for the design. The optical system needs refinement by raytracing (Zemax, Ansys, USA) see Figures S2 and S3 (Supporting Information) and is described in more detail in the section *Raytracing*. This double Scheimpflug design process is also described.^[^ ^35^ ^]^ The optical elements in the spectral analyzer include 3 identical SWIR achromatic lenses, focal length *f* = 75 mm, diameter Ø = 50 mm (AC508‐ 075‐C, Thorlabs, USA), and a 300 groove mm^−1^ transmission grating (GTI50‐03A, Thorlabs, USA). The first achromat, collimates the received light cone, thereafter the grating disperses the light by photon energy. The dispersed light is reimaged onto the camera with the two remaining achromats. The distance between grating and focusing lens is chosen such that the 0th diffraction order can be separated for beam dumping. The axial magnification from slit to camera is 2:1.

The effective magnification of the spectral analyzer unit is 2:1 from slit to 2D detector array. With the raytraced optical system, an opto‐mechanical system was designed with CAD (fusion 360, Autodesk, USA) see Figure S4 (Supporting Information). This is discussed in the below section. The opto‐mechanical system was 3D‐printed in black Polyactic acid (PLA‐DF = 02, Dremel, USA) with a commercial 3D‐printer (3D45, Dremel, USA).

Camera: The camera utilized in the spectral analyzer (Figure 1c) is a 640 × 512 pixel InGaAs camera (C‐RED3, FirstLight, France), with a pixel pitch of 15 × 15 µm and a dynamic range of 14 bit. The spectral range of the sensor is Δ*λ* ≈ 800 nm (900–1700 nm), with a max framerate of 600 fps. The output of the camera is a USB 3.1 Gen 1 port.

Data storage: The hyperspectral lidar is recording backscattered light intensity, *I*(*pix_r,_ pix_*λ*_*), (14 bit) as a function of range (1 ≤ *pix_r_* ≤ 640 pixels) and photon energy (1 ≤ *pix_*λ*_* ≤ 512 pixels) in single exposures of the 2D‐camera detector. The time is recorded by stacking consecutive exposures. The acquisition rate for the free flying insect signals was 90 GB h^−1^, the temporal fill factor was 8% (time gaps between datafiles are caused by real time visualization and saving to hard drives). The hyperspectral images are stored in data cubes where the axes are range, wavelength, and time, respectively. The data are transferred from the camera to a computer by USB 3.1 where an acquisition script (LabView, National Instruments) visualizes the hyperspectral data real time and stores them as raw files with a time stamp.

### Raytracing Design

Raytracing is a numerical simulation of light rays for optimizing performance and optical systems. Raytracing features a merit function tool that allowed optimizing the system with the goal of achieving the smallest spot size in range‐, *δr*, and spectral, *δλ*, domain. The parameters that were adjusted by the merit function are lens separations, position and 2 axis tilt of the detector. A weight function was introduced to optimize the system to perform better in the far range since the pixel footprints increase with range, and thus the impact of a large spot size is greatest at far distances. The result of the optimization can be observed in the spot‐diagram in Figures S2a and S3a,b (Supporting Information) where the root‐mean‐square (RMS, *δr*, *δλ*) is plotted as a function of wavelength for three different range positions. The raytracing predicts a spot size in the spectral domain of ≈40 µm for mid‐ and far range, whereas the spectral spot size at near range is close to the 100 µm image of the demagnified slit (by which the PSF will be convolved with). Thus, raytracing suggests that spectral resolution is constrained by the slit width. The demagnified image of the slit fits ≈76 times across the chip, consequently 76 independent lidar spectral bands can be expected between 1000 and 1600 nm and accordingly a spectral resolution of 8 nm. In addition, binning several spectral pixels would not deteriorate the spectral resolution. In the range domain (Figure S3a, Supporting Information), spot sizes are ≈80 µm for close range and <30 µm (2 pixels) for far ranges. This gives 0.4% relative range accuracy. In practice, the range inaccuracy also depends on the lidar beam width, and the precision in field indicates 1%, which is somewhat better than previous Scheimpflug lidars.^[^ ^53^ ^]^

### CAD‐Design

The opto‐mechanical system of the spectral analyzer was designed with CAD (Fusion 360, Autodesk, USA) see Figure S4 (Supporting Information). The unit is based on a 3D‐printed sandwich structure^[^ ^35^ ^]^ combined with cage system elements. Four rods (ERx, Ø = 6 mm, Thorlabs, USA) connect the 3D‐printed structure with the cage cube (CW4, 30 mm, Thorlabs, USA) using two cage plate adapters (LCP4S, 30–60 mm, Thorlabs, USA). The tilted slit is mounted on a rotation stage (B4CRP M, Thorlabs, USA) with a filter holder (FFM1, Thorlabs, USA). The angle of the slit can be adjusted with the rotation stage. In addition to the angle, three adjustment screws allow vertical positioning. Furthermore, the spacing between the cage cube with the slit and the 3D‐printed part can be adjusted to ensure sharp focus from slit to InGaAs camera. When focus is achieved, the rods can be locked with three lateral screws. The two 3D‐printed parts are held together with bolts, combined with threaded brass inserts. This allows disassembling and reassembling the plastic material. The bolts and the rods furthermore act as a reinforcement structure to avoid deformation of the 3D‐printed material. The camera is attached to the 3D‐printed structure with a milled aluminum camera adapter plate. Baffles are included in the design at different positions along the optical path to reduce stray light. Baffles block forward scattered light (snake light), which becomes more significant at grazing angles. Moreover, horn‐like structures^[^ ^56^ ^]^ were included to absorb the light from diffraction orders other than the first. Light from the 0th, 2nd, and −1st‐diffraction order will be trapped in the beam‐dumps and eventually absorbed. The spectral analyzer is installed on the focus stage of the receiver telescope with a cylindrical aluminum adapter.

### Spectral Analyzer Alignment and Characterization

The system is aligned and characterized using a halogen tungsten light bulb as well as a low‐pressure spectral Xenon lamp to illuminate the slit of the spectral analyzer. An integrating sphere coated with BaSO_4_ (Ø = 200 mm, Oriel, USA) was used to uniformly illuminate the entrance slit.^[^ ^35^ ^]^

First, the system was aligned using the halogen light bulb as a light source. The spectral analyzer can be focused by adjusting the spacing between 3D‐print and cage‐cube (see Figure S4, Supporting Information). A transparent plastic foil with a line pattern was placed in the plane of the entrance slit^[^ ^57^, ^58^ ^]^ and the spacing was adjusted until the black lines were sharpest. Figure S5 (Supporting Information) a) shows the black lines of such line pattern. In Figure S5b (Supporting Information), the intensity is plotted along the range, featuring the line pattern. The line pattern in Figure S5ab, Supporting Information) moreover illustrates the optimization of the far range performance where modulation depth is highest. As in all triangulation approaches to ranging, the observation angle (position along the slit, *pix_r_*) relates to distance tangentially. More details on Scheimflug range calibration can be found in literature.^[^ ^52^, ^53^ ^]^

Such relation was used and Scheimpflug range calibration for the case of 2D detectors of the hyperspectral variety was expanded. The estimated range becomes

(1) $$\hat{r}\left(p_{r},p_{\lambda }\right)=\ell_{\mathrm{BL}}\cot\left(\Phi_{\mathrm{slant}}+\theta_{\mathrm{FoV}}p_{r}+\varphi_{\mathrm{shift}}p_{\lambda }+\varphi_{\mathrm{fan}}p_{\lambda }p_{r}\right)$$

here, *p_r_* and *p_*λ*_* denote the chip normalized pixel positions (values from 0 to 1 across the array detector chip), *ℓ*_BL_ is the length of the baseline (0.814 m), *Ф*_slant_ is the angle between the optical axis of the beam expander and receiver, *θ*_FoV_ is the field of view (±) of the receiver, whereas *φ*_shift_ and *φ*_fan_ denote undesirable wavelength dependent artifacts. The rulings across the slit in Figure S5a (Supporting Information) is used to select 21 pairs of *p_r_* and *p_*λ*_* across the detector array, and fitted the four coefficients; *Ф*_slant_, *θ*_FoV_, *φ*_shift_, and *φ*_fan_. Equation (1) explained the pairs with an *R* ^2^_adj_. = 99.99%. The coefficients are provided in Figure S5d (Supporting Information) along with the 95% confidence interval. As the table indicates, the artifacts are less than 1% across the chip and not significantly different from zero (indicative that artefacts are absent in the ranging domain). More information regarding keystone and smile artifacts in hyperspectral instruments is provided.^[^ ^59^ ^]^

In Figures S6 and S7 (Supporting Information), the halogen tungsten filament lamp was also used as a light source to characterize the spectral sensitivity and flat‐field of the system. The light bulb is presumed to be a blackbody radiator at 3000 K. In Figure S6 (Supporting Information), the measured spectrum of the halogen filament is divided by Plank's radiation spectrum of a 3000 K radiation, resulting in the instrument spectral sensitivity curve. The decrease in sensitivity for longer wavelengths is due to the decrease in the efficiency of the diffraction grating. The camera responsivity slightly attenuate shorter wavelengths. Moreover, dips at ≈1200 and ≈1400 nm are present in the sensitivity curve. The dip around 1400 nm is caused by the BaSO_4_ coating of the integrating sphere which exhibits a dip in reflectance at ≈1400 nm, whereas residual OH ions in the quartz envelope of the halogen bulb account for absorption around both 1200 and 1400 nm. Hence, the dips arose during the calibration and will not limit the performance of the system in field.

In Figure S7a (Supporting Information), the spectral sensitivity is plotted at three different range positions. Figure S7b (Supporting Information) shows a detector exposure with the slit uniformly illuminated. The flat‐field plot in Figure S7c (Supporting Information) describes the sensitivity as a function of range. In practice, the lidar sensitivity range dependence, known as the form factor,^[^ ^60^, ^61^ ^]^ is a complicated matter of overlap between beam and field‐of‐view as well as the environmental conditions (humidity and atmospheric attenuation coefficients). The lower sensitivity at far and near might be caused by partly shielding, because the illumination cone from the integrating sphere is wider than the F/4 cone from the Newton receiver. Therefore, this effect does not necessarily affect field performance.

In Figure S8 (Supporting Information) a low‐pressure spectral Xenon lamp was used to calibrate the system. Figure S8a (Supporting Information) shows a single exposure of the Xenon emission lines recorded with the spectral analyzer. The visibility is enhanced by a high‐pass image filter (Matlab, MathWorks, USA). Figure S8c (Supporting Information) displays the Xenon's atomic emission lines as a function of *pix_*λ*_* around *pix_r_* = 50. Several lines could be identified and literature values were assigned.^[^ ^62^ ^]^ The spectral lines display some artifacts but as in Equation (1), the wavelength can be estimated accordingly

(2) $$\hat{\lambda }\left(p_{r},p_{\lambda }\right)=\lambda_{0}+\lambda_{\mathrm{span}}p_{\lambda }+\delta_{\mathrm{bend}}p_{r}^{2}+\delta_{\mathrm{fan}}p_{\lambda }p_{r}$$

here *p_r_* and *p_*λ*_* again denote the chip normalized pixel positions, *λ*_0_ is the shortest spectral band, *λ*_span_ is the spectral range of the instrument and *δ*_bend_ and *δ*_fan_ are undesired artifacts. The *δ*_bend_ and *δ*_fan_ parameters describe the spectral misregistration caused by the smile effect.^[^ ^59^ ^]^ 17 pairs of *p_r_* and *p_*λ*_* were selected across the chip, and the hyperplane was fitted to the table values (*R* ^2^_adj_ = 99.99%). The fitted parameters are given in Figure S8d (Supporting Information) along with the confidence intervals. In terms of spectral calibration, the two artifacts constitute 2% and 4% across the whole chip. The linewidth (FWHM) in Figure S8c (Supporting Information) of the spectral line at 1473.3 nm is 8 pixels which corresponds to a spectral resolution of Δ*λ* ≈ 9.5 nm. This implies 64 independent effective spectral bands of the system which is much beyond any other elastic lidar system reported.

### Field Calibration of Reflectance

With the collected backscattered light, *I*(*pix_r_,pix_*λ*_*), the reflectance of the target, *R*, was calculated (Figure 2d) as follows

(3) $$\begin{array}{c} R\left(p_{r\_\mathrm{target}},p_{\lambda }\right)=R_{\mathrm{term}}\frac{\left[\begin{array}{c} \left(I_{\mathrm{target}}\left(p_{r\_\mathrm{target}},p_{\lambda }\right)-I_{\mathrm{dark}}\left(p_{r\_\mathrm{target}},p_{\lambda }\right)\right)\\ \;{\hat{r}}^{2}\left(p_{r\_\mathrm{target}},p_{\lambda }\right)S\left(p_{r\_\mathrm{term}},p_{\lambda }\right) \end{array}\right]}{\left[\begin{array}{c} \left(I_{\mathrm{term}}\left(p_{r\_\mathrm{term}},p_{\lambda }\right)-I_{\mathrm{dark}}\left(p_{r\_\mathrm{term}},p_{\lambda }\right)\right)\\ \;{\hat{r}}^{2}\left(p_{r\_\mathrm{term}},p_{\lambda }\right)S\left(p_{r\_\mathrm{target}},p_{\lambda }\right)\eta \end{array}\right]} \end{array}$$

here *R* is the reflectance of a target at pixel position *p_r_* and *p_*λ*_* (associated with the range and photon energy given by Equations (1) and (2). *R*_term_ is the reflectance of the beam termination, which in the case is a black Neoprene foam sheet with a spectrally flat reflectance of 2%. *I*_target_ denotes the measured backscattered intensity from a target at *p*_r_target_ in a spectral band at *p_*λ*_*. *I*_dark_ is the background intensity. Due to environmental structures and structured dark current, *I*_dark_ is pixel specific. *I*_dark_ was measured by manually blocking the beam, this was done several times during the evening since both ambient light and dark current decreases (due to falling temperatures). *I*_term_ is the backscattered light from the beam termination with known reflectance. *S* denotes the instrument sensitivity as a function of range and photon energy. This factor could compensate for distinct spectral sensitivities at the target‐ and termination ranges, but as seen in Figure S7a (Supporting Information), the spectral sensitivity does not change noteworthy. Regarding range dependent sensitivity, the form factor can be expected to be comparable to similar single band systems.^[^ ^61^ ^]^ In addition, InGaAs sensors can suffer from a structured pixel specific gain pattern which is also included in *S*. For the purpose, it is assumed that *S* = 1. Omitting the form factor can imply that the absolute reflectance value is inaccurate. However, as it can be understood from the squared range factors in Equation (3), the expression is valid for diffuse targets, whereas a specular target is presented. Studies of specular lidar targets are rare^[^ ^38^ ^]^ and would require a detailed description of the scattering phase function. The last parameter, *η*, denotes the beam‐target overlap. This factor can be estimated by the relative reduction of the termination echo when the target is in the beam. In the case *η ≈*11%. With these arguments, it is understood that the absolute reflectance value cannot be expected to be accurate.

### Hyperspectral Imaging

A reference hyperspectral measurement^[^ ^16^ ^]^ of the same damselfly wing was carried out in a laboratory with a polarimetric short‐wave infrared (SWIR) camera (HySpex, Norsk Elektro Optikk AS, Norway) with 288 spectral bands (from 0.95 to 2.5 µm). A 150 W broadband halogen lamp was used to illuminate the wing in a specular condition (the illumination and camera axis were at ±56° to wing surface normal), the light was horizontally polarized with an ultra‐broadband polarizer (Meadowlark Optics, USA). A second ultra‐broadband polarizer was used to select and capture the polarimetric properties of the wing. The camera objective had an aperture of Ø  =  25.4 mm and a working distance of 8 cm. The damselfly wing was taped to an index card paper (taped near the axillary area of the wing, see Figure 3a. The wing was then mounted on a black neoprene sheet during the hyperspectral imaging to reduce the background reflection. The captured hyperspectral image of the wing was then calibrated to a diffused reflectance standard with 50% reflectance (Spectralon, LabSphere Inc., USA).

### Fringe Model Hyperspectral Imaging

A fringe model was developed based on the Fresnel equations and thin‐film physics^[^ ^26^ ^]^ to estimate the wing thickness of captured fringes from both EHSL and hyperspectral imaging

(4) $$F\left(\lambda ,d\right)=\frac{4R_{s}\sin^{2}\left(2\pi d\sqrt{n^{2}-\sin^{2}\theta }/\lambda \right)}{{\left(1-R_{s}\right)}^{2}+4R_{s}\sin^{2}\left(2\pi d\sqrt{n^{2}-\sin^{2}\theta }/\lambda \right)}$$

where *λ* is the wavelength, *d* is the thickness of the wing, *θ* is the incident angle to the membrane (56° for laboratory recordings and 0.25° for EHSL). *R*_s_ is the reflection coefficient according to Fresnel equations, and *n* is the refractive index of the wing membrane

(5) $$R_{s}={\left(\frac{\cos\theta -\sqrt{n^{2}-\sin^{2}\theta }}{\cos\theta +\sqrt{n^{2}-\sin^{2}\theta }}\right)}^{2}$$

and the refractive index of the chitin in the wing membrane is expressed as^25^

(6) $$n=A+B/\lambda^{2}$$

where *A*  =  1.517 and *B*  =  8800 nm^2^.

For the hyperspectral imaging (Figure 2), the fringe model *F*(*λ,d*) was used to generate multiple (1000x) computed fringes with wing thickness in between range 0.35–4 *µ*m. The modulation of computed fringes *F*(*λ,d*) are always 100% since there are no bias terms. To determine the thickness of the captured fringe with the reflectance *R*(*λ*) from the hyperspectral imaging, each computed fringe *F*(*λ,d*) was then compared to the captured fringe *R*(*λ*) based on correlation, *C* in Equation (7) with a fitting quality parameter, *Q* in Equation (8)

(7) $$\begin{array}{c} C\left(R,F\left(d\right)\right)=\frac{\int_{0.35}^{4}\left(F_{\lambda ,d}-\mu \left(F_{\lambda ,d}\right)\right)\left(R_{\lambda }-\mu \left(R_{\lambda }\right)\right)}{\sqrt[{2}]{\int_{0.35}^{4}{\left(F_{\lambda ,d}-\mu \left(F_{\lambda ,d}\right)\right)}^{2}\partial \lambda \int_{0.35}^{4}{\left(R_{\lambda }-\mu \left(R_{\lambda }\right)\right)}^{2}\partial \lambda }} \end{array}$$

(8) $$Q\left(d\right)=C\left(R,F\right){\left(C\left(\frac{\partial R}{\partial \lambda },\frac{\partial F}{\partial \lambda }\right)\right)}^{2}$$

where *R* is the measured reflectance and *F* is the computed fringe. Here, the spectral derivatives are used to ignore slope differences. The squared power is needed to avoid double negative correlation coefficients. The modulation depth of the captured fringe can be determined by

(9) $$M=\frac{\sigma \left(R_{\lambda }\right)\cdot \mu \left(F_{\lambda }\right)}{\sigma \left(F_{\lambda }\right)\cdot \mu \left(R_{\lambda }\right)}$$

*σ* is the standard deviation, and *µ* is the mean of the spectra. As the examples in Figure 3e show, effective fringe amplitudes increase by wavelength because the narrower fringes at shorter wavelengths are more prone to dephase and interfere destructively due to thickness heterogeneities across the wing surface.

### Fringe Model Hyperspectral Lidar

In order to match the increasing amplitude of the hyperspectral lidar measurements, the fringe model *F*(*λ,d*) with a long pass function is multiplied to obtain a wing reflectance model

(10) $$R_{\mathrm{wing}}\left(d\right)=\frac{{\left(\frac{\lambda }{\lambda_{0}}\right)}^{\alpha }}{1+{\left(\frac{\lambda }{\lambda_{0}}\right)}^{\alpha }}\cdot \left(R_{\mathrm{bias}}+R_{\mathrm{fringe}}\cdot F\left(\lambda ,d\right)\right)$$

here *λ*_0_ is the cut‐on wavelength for the long pass function and *α* is the long pass steepness. Since thickness heterogeneities across the wing damp the effective wing fringe toward shorter wavelengths, *λ*_0_ is expected to scale with this heterogeneity. Other phenomena, such as gradient refractive coatings in damselflies could also contribute to this effect.^[^ ^25^ ^]^ *R*_bias_ is a scalar indicating a nonfringy bias of the spectrum. *R*_fringe_ is a scalar indicating the amplitude of the effective fringe.

The parameters; *λ*_0_, *α, R*_bias_, *R*_fringe_, and *d* were fitted to the measured and calibrated reflectance from the damselfly wing target, *R*, from Equation (3). The fit was done using a numerical search algorithm (Curve Fitting Toolbox, Matlab, MathWorks, USA), and ended at *R* ^2^_adj_ 96% with the following values (**Table** **1**).

**Table 1:** Fitting parameters for thin‐film model

Parameter	Value	Confidence interval	Unit
*d*	1412	[1408, 1416]	nm
*λ*_0_	1419	[1370, 1469]	nm
*α*	7.7	[7.0, 8.4]	
*R*_bias_	14	[12, 15]	%
*R*_fringe_	111	[99, 123]	%

## Conflict of Interest

The authors declare no conflict of interest.

## Author Contributions

M.B. and N.R. acquired grants for salaries and materials, L.M., M.L., A.R., and M.B., conceived and designed study, A.R. selected species, M.L. prepared specimen, L.M., H.M., J.S., and M.B. designed, and built the lidar, L.M., M.L., H.M., J.S., N.R., C.K., A.R., and M.B. participated in field work, M.L., J.H., M.B. contributed hyperspectral laboratory recordings, L.M., M.L., and M.B. analysis code, L.M. produced all graphics with M.B. supervision. L.M., M.L., and M.B. drafted the manuscript, all authors contributed to the manuscript.

## Supporting information

Supporting Information

## Data Availability

The data that support the findings of this study are available from the corresponding author upon reasonable request.
