# Supplementary material for: Remote Nanoscopy with Infrared Elastic Hyperspectral Lidar
Source: Adv Sci (Weinh). 2023 Mar 25;10(15):2207110. doi: 10.1002/advs.202207110 (PMC10214245; doi:10.1002/advs.202207110)
Supplement: Supplementary file 1 — Supporting Information [file ADVS-10-2207110-s001.pdf]

Supplementary Information for:

Remote Nanoscopy with Infrared Elastic Hyperspectral Lidar

Lauro Müller[1*], Meng Li[1], Hampus Månefjord[1], Jacobo Salvador[1], Nina Reistad[1,2], Julio Hernandez[3], Carsten Kirkeby[4,5], Anna Runemark[6], Mikkel Brydegaard[1,3,5,6]

[1] Dept. Physics, Lund University, Sölvegatan 14c, 22363 Lund, Sweden

[2] Centre for Environmental and Climate Science, Lund University, Sölvegatan 37, SE-223 62 Lund, Sweden

[3] Norsk Elektro Optikk A/S, Østensjøveien 34, 0667 Oslo, Norway

[4] Dept. Veterinary and Animal Sciences, Copenhagen University, 1870 Frederiksberg, Denmark

[5] FaunaPhotonics, Støberigade 14, 2450 Copenhagen, Denmark

[6] Dept. Biology, Lund University, Sölvegatan 35, 22362 Lund, Sweden

* correspondence: lauro.muller@forbrf.lth.se

This PDF file includes:

Supplementary Fig. s S1 to S8

Supplementary figures:

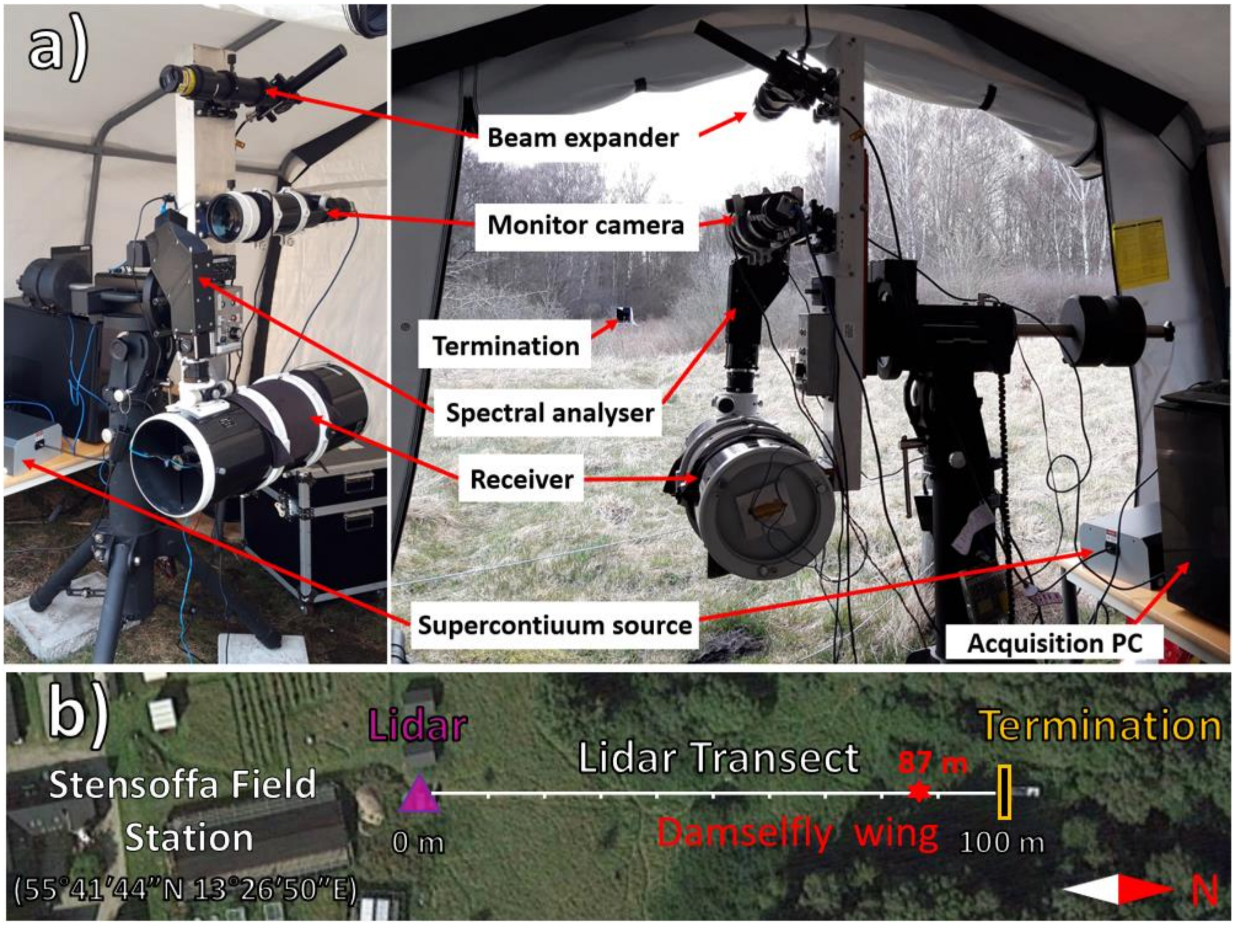

Fig. S1: Field site. a) Fully mounted EHSL pointing at the termination during field measurements at Stensoffa field station, April 2022. b) Aerial view of the lidar site in Stensoffa.

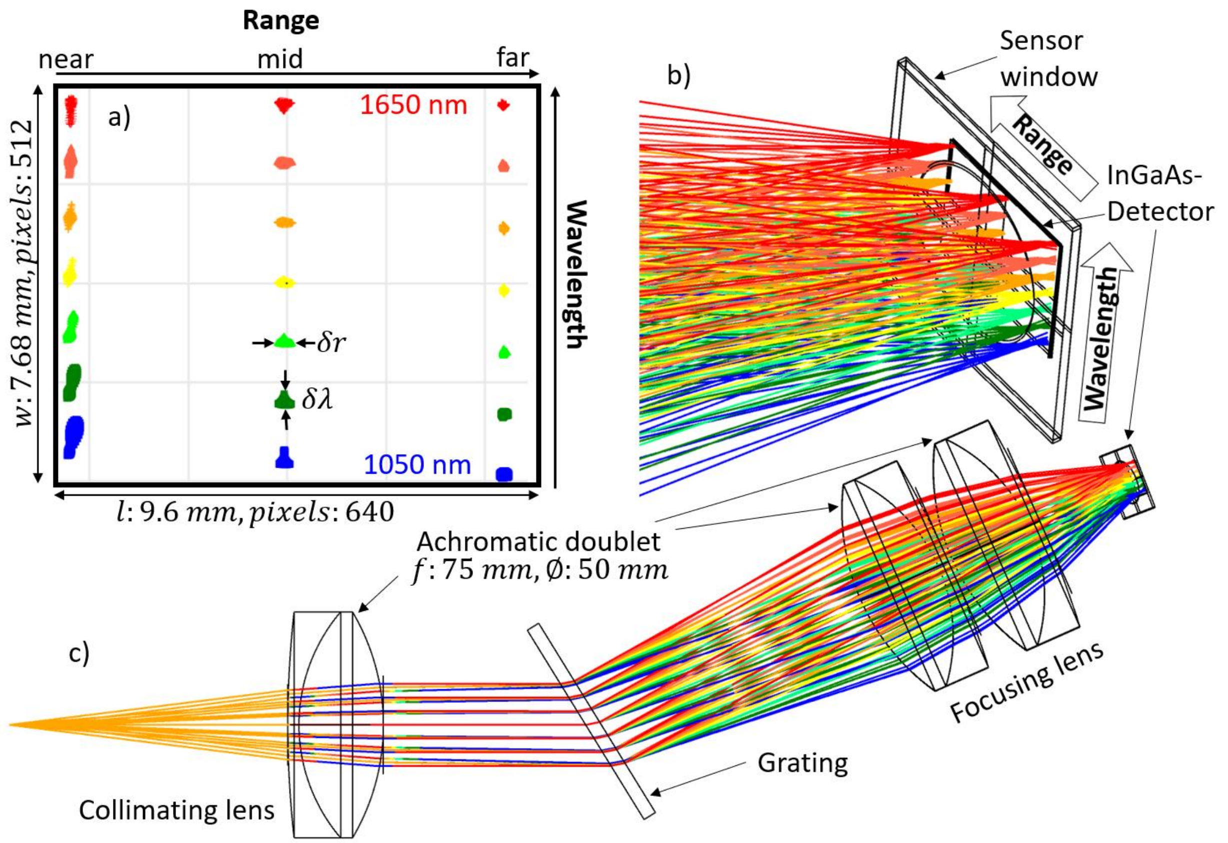

Fig. S2: Raytracing simulation of the spectral analyzer. c) Simulation how the spectrum is determined. b) Rotated close-up view of the detector. a) Spot size diagram. The system is optimized to perform best in the far range which can be seen in the spot diagram.

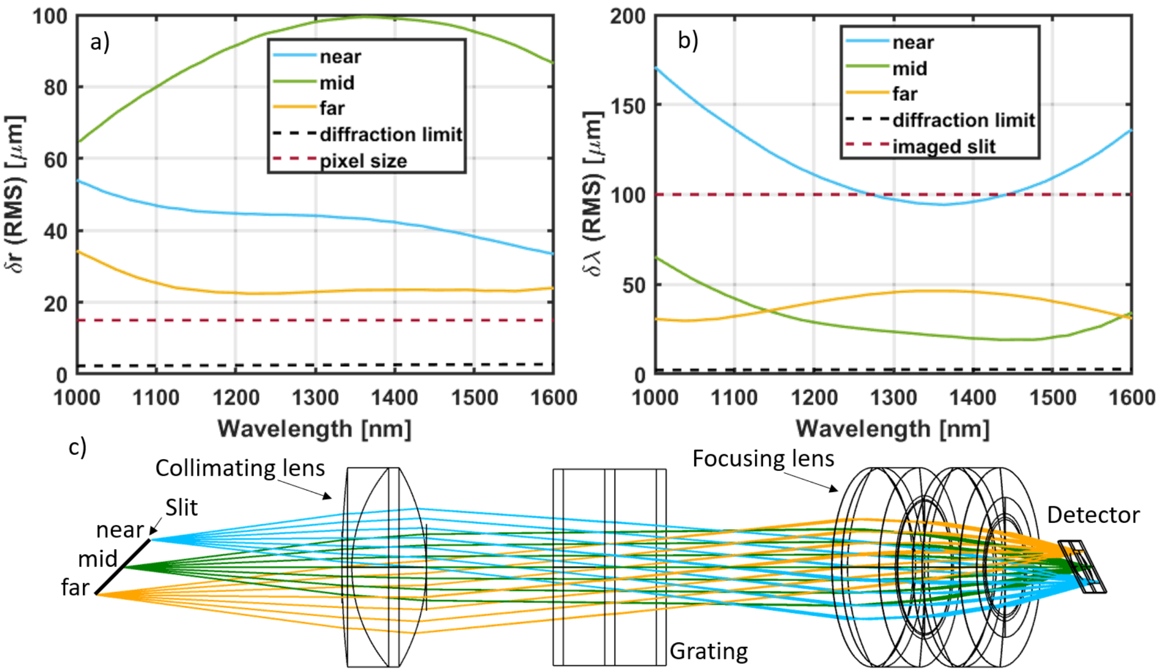

Fig. S3: Raytracing simulation of the spectral analyzer. ab) RMS values of the spot size diagram in Fig S2a), plotted as a function of wavelength at different range positions. Note that the values for δr and $\delta\lambda$, are referred to the spot size on the detector and are not range and spectral resolutions of the system. c) Simulation of how range is determined.

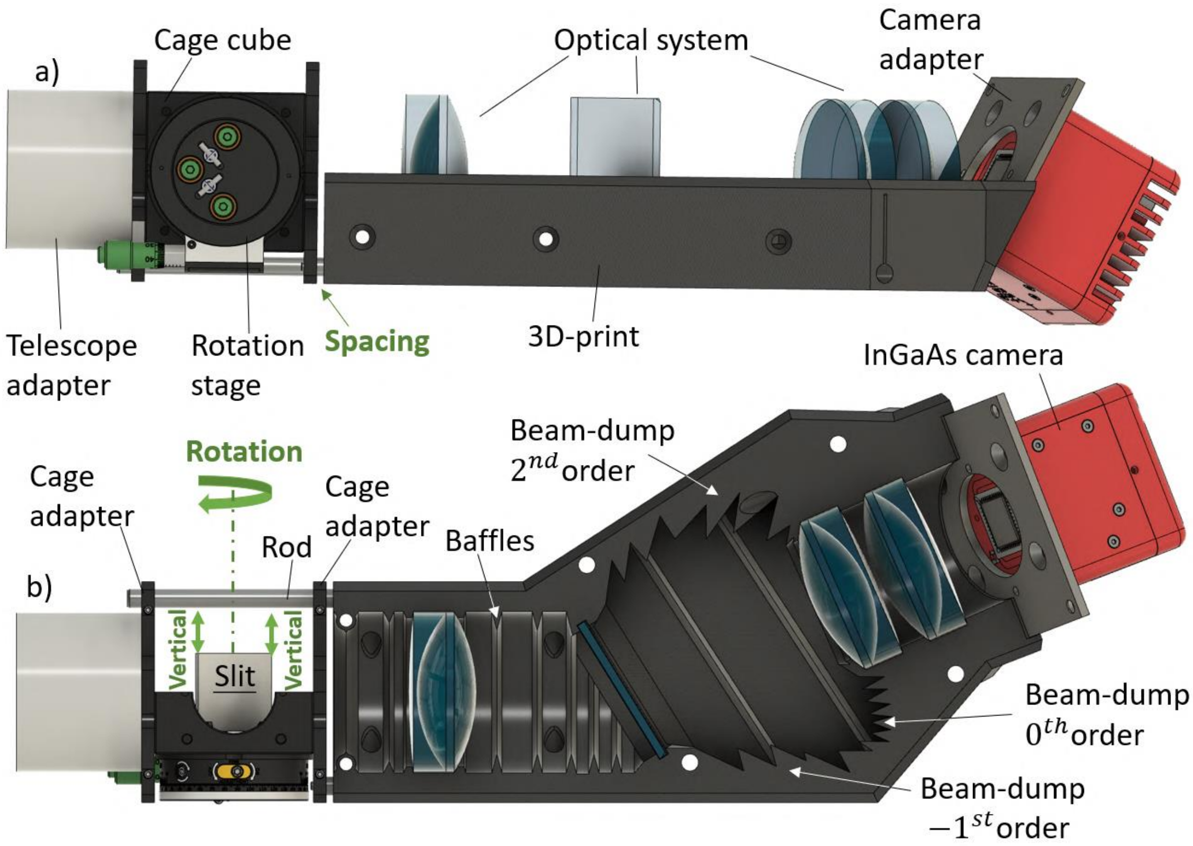

Fig. S4: a) Sideview and b) topview of the spectral analyzer. Two 3D-printed bodies form together a sandwich structure (only the bottom is depicted) in which the optics is enclosed. The 3D-printed structure is complemented with standard off-the-shelf components. The degrees of freedom for alignment are highlighted in green.

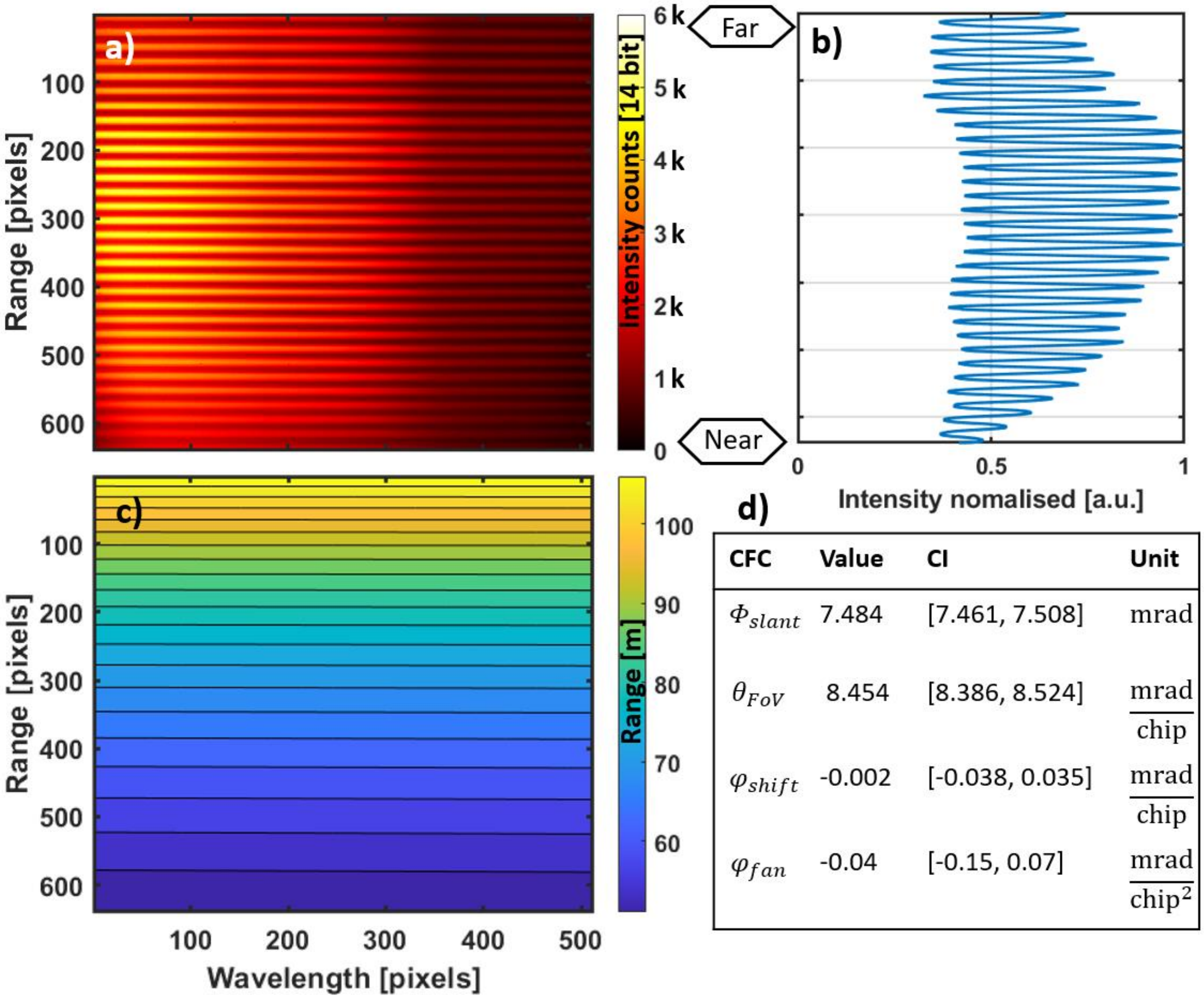

Fig. S5: a) Detector-image with line pattern in front of the entrance slit to adjust focus, using a halogen bulb as a light source. b) Intensity plotted as a function of range. Note that the system is optimized to perform best in the far range. c) Hyperplane to convert pixel into range in units of meters. d) Fitted parameters and artifacts with confidence intervals.

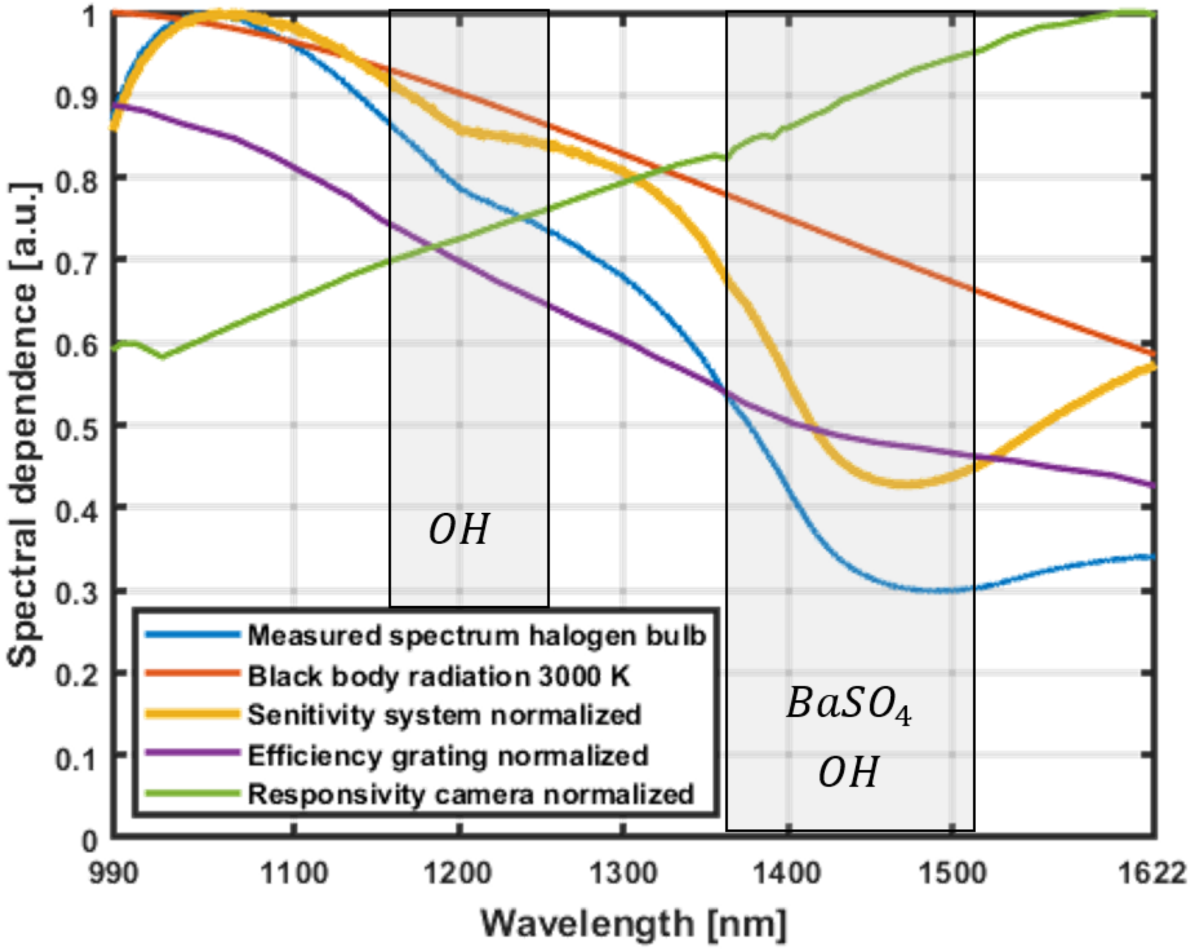

Fig. S6: Spectral sensitivity plot of the EHSL, using the measured blackbody radiation of a halogen bulb (3000 K) which is compared to the theoretical black body radiation at 3000 K. The grating efficiency and the camera responsivity are plotted as well. The dips appearing in the sensitivity plot at ≈ 1200 nm and ≈ 1400 nm are caused by the $BaSO_4$ coating of the integrating sphere and by OH from the quartz glass of the halogen bulb, respectively.

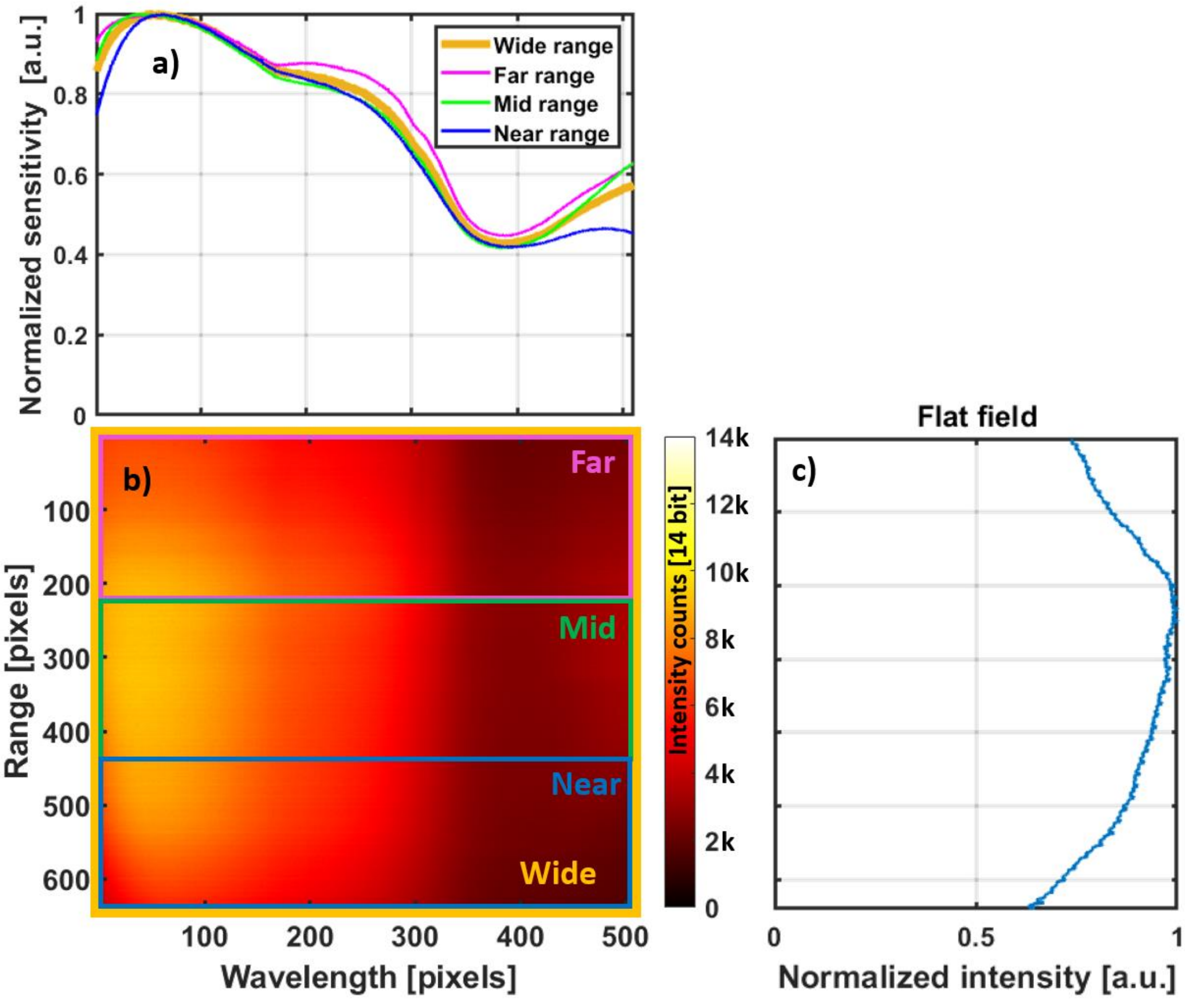

Fig. S7: a) Spectral sensitivity plot of the EHSL, at three different range positions using the blackbody radiation of a halogen bulb. b) Detector-image with uniformly illuminated slit. c) Flat-field which is the intensity as a function of range. For a uniform illumination, the flat-field is expected to feature a top-hat profile.

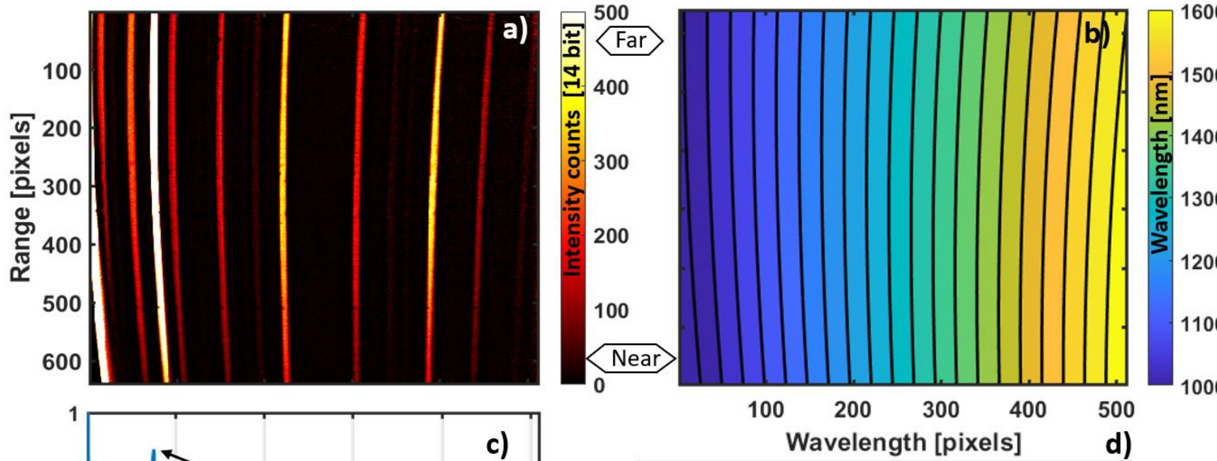

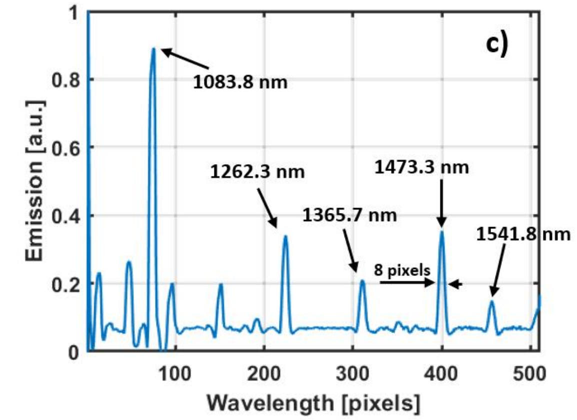

d)

Coeff	Value	CI	Unit
λ_0	995.3	[988.5, 1002]	nm
λ_{span}	607.3	[-594.7, 619.9]	$\frac{nm}{chip}$
bend	-25.23	[-37.18, -13.29]	$\frac{nm}{chip^2}$
fan	57.1	[37.13, 77.07]	$\frac{nm}{chip^2}$

Fig. S8: Spectral calibration using a Xenon-lamp. a) Spectral emission lines of Xe recorded with EHSL. b) Hyperplane to convert pixel into wavelength at different range positions. c) Spectrum in the far range with the assigned values in units of nm. d) Coefficients and artifacts from the hyperplane spectral calibration model after fitting to multiple points across the sensor array.
